# Integrated Single-Cell RNA-Sequencing Analysis of Aquaporin 5-Expressing Mouse Lung Epithelial Cells Identifies GPRC5A as a Novel Validated Type I Cell Surface Marker

**DOI:** 10.3390/cells9112460

**Published:** 2020-11-11

**Authors:** Masafumi Horie, Alessandra Castaldi, Mitsuhiro Sunohara, Hongjun Wang, Yanbin Ji, Yixin Liu, Fan Li, Thomas A. Wilkinson, Long Hung, Hua Shen, Hidenori Kage, Ite A. Offringa, Crystal N. Marconett, Per Flodby, Beiyun Zhou, Zea Borok

**Affiliations:** 1 Hastings Center for Pulmonary Research and Division of Pulmonary, Critical Care and Sleep Medicine, Department of Medicine, Keck School of Medicine, University of Southern California, Los Angeles, CA 90033, USA; mhorie-tky@umin.ac.jp (M.H.); acastald@usc.edu (A.C.); mitsuhiro.sunohara@nifty.ne.jp (M.S.); hjwang@chla.usc.edu (H.W.); yanbinji@usc.edu (Y.J.); yixinliu@usc.edu (Y.L.); huashen@usc.edu (H.S.); pflodby@hotmail.com (P.F.); bzhou@usc.edu (B.Z.); 2 Department of Respiratory Medicine, Graduate School of Medicine, The University of Tokyo, 7-3-1 Hongo, Bunkyo-ku, Tokyo 113-0033, Japan; kageh-tky@umin.ac.jp; 3 Division for Health Service Promotion, The University of Tokyo, 7-3-1 Hongo, Bunkyo-ku, Tokyo 113-0033, Japan; 4 Single-Cell, Sequencing, and CyTOF Core (SC2), Children’s Hospital Los Angeles, Los Angeles, CA 90027, USA; fanli.gcb@gmail.com (F.L.); tomwilkinson2010@gmail.com (T.A.W.); lhung@chla.usc.edu (L.H.); 5 Department of Surgery, Keck School of Medicine, University of Southern California, Los Angeles, CA 90033, USA; ilaird@usc.edu (I.A.O.); cmarcone@usc.edu (C.N.M.); 6 USC Norris Comprehensive Cancer Center, Keck School of Medicine, University of Southern California, Los Angeles, CA 90033, USA; 7 Department of Biochemistry and Molecular Medicine, Keck School of Medicine, University of Southern California, Los Angeles, CA 90033, USA

**Keywords:** aquaporin 5 (AQP5), alveolar epithelial type 1 cell, scRNA-seq, GPRC5A, AT1 cell marker

## Abstract

Molecular and functional characterization of alveolar epithelial type I (AT1) cells has been challenging due to difficulty in isolating sufficient numbers of viable cells. Here we performed single-cell RNA-sequencing (scRNA-seq) of tdTomato^+^ cells from lungs of AT1 cell-specific Aqp5-Cre-IRES-DsRed (ACID);R26tdTomato reporter mice. Following enzymatic digestion, CD31^-^CD45^-^E-cadherin^+^tdTomato^+^ cells were subjected to fluorescence-activated cell sorting (FACS) followed by scRNA-seq. Cell identity was confirmed by immunofluorescence using cell type-specific antibodies. After quality control, 92 cells were analyzed. Most cells expressed ‘conventional’ AT1 cell markers (*Aqp5*, *Pdpn*, *Hopx*, *Ager*), with heterogeneous expression within this population. The remaining cells expressed AT2, club, basal or ciliated cell markers. Integration with public datasets identified three robust AT1 cell- and lung-enriched genes, *Ager*, *Rtkn2* and *Gprc5a*, that were conserved across species. GPRC5A co-localized with HOPX and was not expressed in AT2 or airway cells in mouse, rat and human lung. GPRC5A co-localized with AQP5 but not pro-SPC or CC10 in mouse lung epithelial cell cytospins. We enriched mouse AT1 cells to perform molecular phenotyping using scRNA-seq. Further characterization of putative AT1 cell-enriched genes revealed GPRC5A as a conserved AT1 cell surface marker that may be useful for AT1 cell isolation.

## 1. Introduction

The lung alveolar epithelium comprises two morphologically and functionally distinct cell types: type I (AT1) and type II (AT2) cells. AT2 cells cover ~5% of the alveolar surface and have important functions including the production of surfactant proteins and both self-renewal and differentiation to AT1 cells during homeostasis and repair following injury [1,2,3,4]. AT1 cells are large, flat cells with long cytoplasmic processes that cover more than 95% of the alveolar surface and play a key role in gas exchange and ion transport [5,6]. In contrast to AT2 cells, which have been extensively studied, far less is known about AT1 cell contributions to alveolar homeostasis due to difficulty in isolating viable AT1 cell populations of sufficient yield and purity for detailed molecular and functional characterization.

An in vitro culture model which recapitulates aspects of AT2 to AT1 cell transdifferentiation has provided important insights into functional and phenotypic properties of AT1 cells [7,8,9,10]. While transdifferentiated ‘AT1-like’ cells express many of the phenotypic markers characteristic of AT1 cells in situ, and their transcriptomic profile overlaps considerably with that of freshly isolated AT1 cells, they are likely not entirely identical to AT1 cells due to alterations occurring in culture [11,12], making it important to validate in vitro findings using freshly isolated AT1 cells. Isolation of viable AT1 cells has been challenging due to their fragility based on their topological complexity, while lack of specific AT1 cell surface markers for fluorescence-activated cell sorting (FACS) or magnetic-activated cell sorting (MACS) has limited the ability to enrich for them [5,12,13].

Aquaporin 5 (AQP5), podoplanin (PDPN), homeodomain-only protein (HOPX) and more recently advanced glycation end products receptor (AGER) have been viewed as ‘classical’ AT1 cell markers [4,14]. However, all have limitations with regard to specificity for AT1 cells within the lung and are also expressed at other extrapulmonary sites, limiting their utility for isolation of pure populations of AT1 cells and generation of AT1 cell-specific Cre driver lines. In this regard, the use of podoplanin (PDPN) for AT1 cell isolation has been reported, but PDPN is also expressed in bronchial and lymphatic cells in the lung and failed to separate ATI cells from bronchiolar epithelial cells [15]. We recently identified by transcriptome profiling of rat AT1 and rat and human AT1-like cells a number of putative novel AT1 cell markers, including the GRAM domain 2 (GRAMD2) gene [12]. However, further validation of a panel of robust murine AT1 cell markers is needed for the development of novel tools for characterization of AT1 cell molecular phenotype.

Single-cell RNA-sequencing (scRNA-seq) is a powerful technique enabling characterization of the molecular states of individual cells by analyzing their transcriptional profiles. Implementation of this technique has advanced our understanding of cellular heterogeneity in various tissues and disease states [16,17,18,19,20]. In scRNA-seq analyses of whole lung, AT1 cells have been captured to only a limited extent [21,22,23], and the proportion of AT1 cells recovered for analysis has been quite low compared to that of other cell types. We therefore considered enriching for AT1 cells prior to scRNA-seq as a potential approach to facilitate further characterization of AT1 cell transcriptomes.

AQP5 is a water channel protein that is expressed on the apical surface of AT1 cells of the alveolar epithelium [24,25,26]. It is also expressed on some cells in the large airways, and as previously reported, depending on the strain of mice, on a small subset of AT2 cells [25]. We previously generated *Aqp5-Cre-IRES-DsRed* (ACID) mice in which a *Cre-IRES-DsRed* cassette was inserted into exon 1 of the endogenous *Aqp5* gene, which is abundantly expressed in AT1 cells. However, ACID mice did not show a specific DsRed signal, likely due to low levels of protein expression. Thus, in the present study ACID mice were crossed with *ROSA26-stop^flox^-tdTomato* conditional knockin mice, resulting in *ACID;R26tdTomato* mice that express tdTomato in *Aqp5*-expressing cells. The tdTomato^+^ population included cells identified as AT1 cells as well as other lung cell types; nevertheless, using these *Aqp5*-Cre reporter mice, we obtained sufficient enrichment to successfully perform scRNA-seq of a population of cells identified by Uniform Manifold Approximation and Projection (UMAP) as AT1 cells. Consistent with our previous study [27], analysis of the expression pattern of ‘classical’ AT1 cell markers within this AT1 cell population at a single-cell level revealed considerable heterogeneity. By integrating our data with public datasets [28,29,30,31] and a previous dataset derived from our in vitro AT2 to AT1 cell differentiation model [12], we identified three conserved lung- and AT1 cell-enriched genes: *Ager*, rhotekin 2 (*Rtkn2*) and G protein-coupled receptor class C group 5 member A (*Gprc5a*). One of these, *Ager*, has previously been identified as enriched in AT1 cells, although it is also expressed in other lung cells [14]. Finally, we evaluated cell-specific protein expression in the airways and distal lung by immunofluorescence staining and validated GPRC5A as a novel AT1 cell surface marker.

## 2. Materials and Methods

### 2.1. Generation of Aqp5-Reporter Mice

To generate AT1 cell-specific reporter mice, *Aqp5-Cre-IRES-DsRed* (ACID) mice in which a *Cre-IRES-DsRed* cassette is knocked into exon 1 of the endogenous *Aqp5* gene were crossed with mice with a *ROSA26-stop^flox^-tdTomato* conditional knockin allele [25,32]. Because our ACID knockin construct which includes the DsRed gene coding for red fluorescent protein showed no specific DsRed signal, likely due to low levels of protein expression, we used *ROSA26-stop^flox^-tdTomato* mice as reporter mice [25]. Double-heterozygous mice (termed *ACID;R26tdTomato*) which express the red fluorescent protein Tomato after Cre/loxP recombination were generated (Supplemental Figure S1). Mice were on an 129S6/SvEvTac background. Three male mice were used for optimization of the digestion and sorting strategy. A four-month old male mouse was used for scRNA-seq analysis.

### 2.2. Lung Digestion

Mouse lungs were surgically harvested following perfusion with phosphate-buffered saline (PBS) via the right ventricle. After digestion with 0.2% pronase (Roche, Indianapolis, IN, USA) and 0.08% collagenase/dispase (Roche), lungs were minced, suspended in Dulbecco’s modified Eagle’s medium (DMEM)/F12 (Sigma, St. Louis, MO, USA) with 0.01% DNase I (Roche), and sequentially filtered through cell strainers of 100 μm and 40 μm pore size (BD Falcon, Franklin Lakes, NJ, USA). Cells were resuspended in Red Blood Cell Lysis Buffer (Roche) for 1 min on ice and washed with PBS. The resulting cell preparation was suspended in cold buffer consisting of PBS with 2% fetal bovine serum (FBS, HyClone/Thermo Fisher Scientific, Tustin, CA, USA), 2 g/L glucose (Sigma) and 20 mM HEPES (Sigma) at a density of 10^6^ cells/100 μL, and subsequently incubated with antibodies (Abs) as described below for cell sorting.

### 2.3. Flow Cytometry

After pre-incubation with Ultra-LEAF purified anti-mouse CD16/32 Ab (Biolegend, San Diego, CA, USA) for 5 min on ice to block of Fc receptors, cells were incubated with primary Abs on ice for 45 min as follows: CD31-biotin (1:50, clone: 390, eBioscience, San Diego, CA, USA), CD45-biotin (1:100, clone: 30-F11, eBioscience) and CD324 (E-Cadherin )-PerCP-eFluor 710 (1:20, Thermo Fisher Scientific). After washing 3 times, cells were incubated with Streptavidin Allophycocyanin (1:200, APC, BD Biosciences, San Jose, CA, USA) secondary Ab for 30 min on ice. After washing 3 times, APC-negative, PerCP-eFluor 710-positive, and tdTomato-positive cells were sorted with a FACSAria cell sorter (BD Biosciences) in preparation for scRNA-seq. Cell viability following FACS was measured using trypan blue vital dye staining, and >95% of cells were confirmed to be viable. To confirm tdTomato expression in sorted cells, 1 × 10^5^ sorted cells were fixed with 4% paraformaldehyde (PFA), and cytospins were prepared by applying cells to EZ Single Cytofunnels (Thermo Fisher Scientific). Cytospins were counterstained with 4’,6-diamidino-2-phenylindole (DAPI, Vector Laboratories, Burlingame, CA, USA), and tdTomato expression was confirmed using a Nikon Eclipse 80i microscope (Nikon Instruments, Inc., Melville, NY, USA).

### 2.4. scRNA-Seq

A single-cell suspension was loaded onto a C1 mRNA Seq HT IFC (High Throughput Integrated Fluidics Circuit, Fluidigm, South San Francisco, CA, USA) designed to capture 800 single cells with diameters of 10–17 µm. The IFC was run on a C1 Single Cell Auto Prep System (Fluidigm) using the C1 Single-Cell mRNA Seq HT Reagent Kit v2 (Fluidigm). After initial cell capture, the IFC was scanned on a Leica DMI6000B microscope (Leica Microsystems, Wetzlar, Germany) to verify cell capture. Cell lysis, reverse transcription, PCR amplification, cell bar code tagging and harvesting were done onboard the C1 System. Harvested sample cleanup was performed using AMPure XP beads (Beckman Coulter, Brea, CA, USA). Sequencing libraries were generated using the Nextera XT DNA Library Preparation Kit (Illumina, San Diego, CA, USA), with indexes from Nextera XT Index Primer Set A and Set B. Libraries were pooled, and library quality was assessed on an Agilent High Sensitivity DNA Chip (Agilent Technologies, Santa Clara, CA, USA). The pooled library, combined with 30% PhiX, was loaded on a Nextseq 500/550 High Output Kit v2 Flowcell (Illumina) and sequenced on a Nextseq 500 Sequencing System (Illumina) using paired end reads (R1–26bp, R2–120bp).

### 2.5. Data Processing

Quality control of sequence reads was performed by Trim Galore and FastQC/multiQC [33]. Mapping was performed by HISAT2 against GRCm38 [34], and quantification of mapped sequence reads was calculated by featureCounts [35]. Quality control of cells was performed using the following parameters: (1) total read counts (>90,000 counts), (2) detected genes (>500 genes) and (3) proportion of sequence reads from mitochondrial genes (<0.15) [36]. Expression level of cell population markers was evaluated as absolute read count. Data clustering, including Uniform Manifold Approximation and Projection (UMAP) and identification of cluster-specific marker genes, was done using R package Seurat v3.0 [37]. Pathway analysis was performed using Ingenuity Pathway Analysis (IPA, QIAGEN, Redwood City, CA, USA). For the analysis of the bulk RNA-seq data obtained from GTEx portal site [31], counts per million were used for normalization between samples. The dataset has been deposited in the GEO database (GSE120285).

### 2.6. Comparison with Public Datasets

For validation of AT1 cell-enriched markers, (1) the scRNA-seq dataset of normal adult mouse lung was obtained from GSE108097 (Mouse Cell Atlas) [28], (2) the scRNA-seq dataset of postnatal day 1 mouse lung was obtained from the LungMAP portal site [29], (3) the scRNA-seq dataset of AT1 cells from postnatal day 60 mouse lung was obtained from GSE106960 [30], (4) bulk RNA-seq data from the human AT2-AT1 cell in vitro differentiation model were obtained from GSE66627 [12] and (5) bulk RNA-seq data of 334 normal lung tissues and 8564 other tissues from 29 different organs were obtained from the GTEx portal site [31]. Localization of protein expression was validated using data from the Human Protein Atlas (https://www.proteinatlas.org/) [38]. For evaluation of *Aqp5* expression in airway cells, the scRNA-seq dataset of airway epithelium was obtained from the Single Cell Portal site (https://portals.broadinstitute.org/single_cell/) [39].

### 2.7. Immunofluorescence Staining of Frozen Lung Sections and Cytospins

For mouse and rat, lungs were cleared of blood by perfusing in PBS, fixed in 4% PFA, incubated in sucrose solution, filled with Optimal Cutting Temperature Compound (OCT; VWR, Radnor, PA, USA))/50% PBS and frozen in OCT. Paraffin-embedded samples were prepared from human lungs that were deemed not suitable for transplantation. Lung cryosections (5 μm) were prepared as described [40]. Following antigen retrieval in Antigen Unmasking Solution at low pH (Vector Laboratories), slides of lung sections were incubated for 30 min in 0.2% Triton-X in PBS to permeabilize cells. After incubation in CAS block (Invitrogen/Zymed, San Diego, CA, USA), slides were incubated with primary Abs overnight at 4 °C. Goat anti-pro-SPC (sc-7706; Santa Cruz Biotechnology, Inc., Santa Cruz, CA, USA), mouse anti-ABCA3 (17-H5-24; Seven Hills, Cincinnati, OH, USA), mouse anti-HOPX (sc-398703; Santa Cruz), goat anti-AQP5 (sc-9890; Santa Cruz), goat anti-CC10 (sc-9772; Santa Cruz) or rabbit anti-GPRC5A (abx005719; Abbexa, Cambridge, UK) were used as primary Abs. Slides were then incubated with biotinylated anti-goat (Millipore, Temecula, CA, USA), biotinylated anti-rabbit (Millipore) or Alexa Fluor 594 anti-mouse (Thermo Fisher Scientific) secondary Abs, followed by streptavidin-FITC (Vector). Normal goat IgG (Vector), normal rabbit IgG (Vector) or normal mouse IgG (Vector) were used as negative controls. Finally, slides were mounted with Vectashield mounting medium including DAPI (Vector). Confocal images were captured using a Leica SP8 confocal system (Leica Microsystems), and negative controls were used to set the laser intensity (Supplemental Figures S7 and S8 and Figures 3C,D, 5A, 6A, and 7). Cytospins of crude cells harvested from digestion of mouse lungs were prepared as described for sorted cells above. For immunostaining, cytospins were post-fixed in 4% PFA, and antigen retrieval and staining were performed as described above for frozen lung sections. The percentage of cells that were double positive for tdTomato and pro-SPC or CC10 in immunofluorescence staining of lung sections was calculated by manually counting 10 random fields, and the percentage of cells that expressed tdTomato in immunofluorescence staining of cytospins was calculated by manually counting 5 random fields. Microscopic evaluation was independently validated by two blinded operators.

### 2.8. Statistics

R package Seurat v3.0 was used for statistical analysis. Spearman’s correlation coefficient (R) was calculated for correlation analysis, and *p* < 0.05 was regarded as significant. For violin plots, the smoothed curves were generated by Seurat using the kernel density estimator.

## 3. Results

### 3.1. Isolation of AT1 Cells from ACID;R26tdTomato Mice

**Figure 1:**
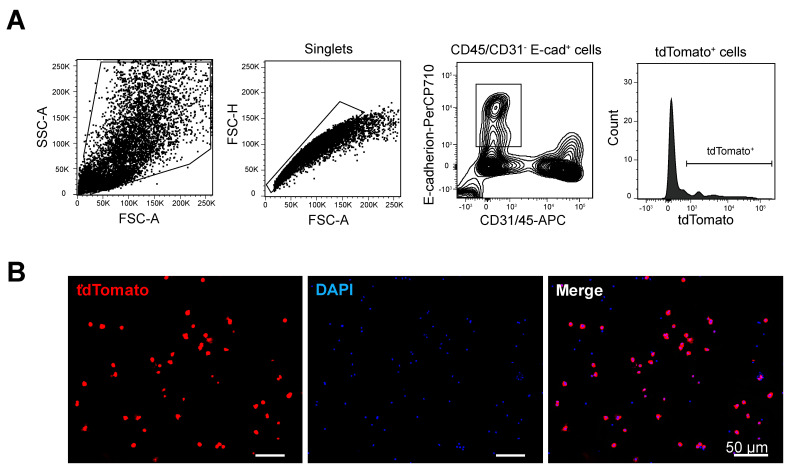
Sorting of tdTomato^+^ cells from *ACID;R26tdTomato* mice. (**A**) Flow cytometry strategy for sorting AT1 cells. CD45/CD31 negative, E-cadherin positive, tdTomato positive cells were sorted; 31% ± 10% (*n* = 3) of epithelial cells from test mice were positive for tdTomato (panel 4). The box in the third panel delineates the CD45/CD31-negative and E-cadherin-positive populations. (**B**) Immunofluorescence images of cytospins after fluorescence-activated cell sorting (FACS). Approximately 80% of cells are positive for tdTomato.

To optimize the lung digestion and sorting strategy, we initially isolated AT1 cells from three *ACID;R26tdTomato* mice that expressed the red fluorescent protein tdTomato in Aqp5-expressing cells (Supplemental Figure S1). Single-cell RNAseq was subsequently performed on sorted cells from one four-month-old male mouse (Figure 1). After enzymatic digestion of *ACID;R26tdTomato* mouse lungs, cells were incubated with CD45/CD31/E-cadherin Abs, and CD31^-^CD45^-^E-cadherin^+^tdTomato^+^ cells were sorted by FACS (Figure 1A, Supplemental Figure S2). FACS analysis of cells isolated from three lungs showed that 31 ± 10% of epithelial cells were positive for tdTomato (data not shown). By fluorescence microscopy, we confirmed that most sorted cells (80%) examined in cytospins were positive for tdTomato (Figure 1B). The remaining cells (~20%) were negative for tdTomato, perhaps due to loss of their cytoplasm as a result of the FACS procedure. A sorted cell suspension was loaded onto the C1 Fluidigm system, and manual checking by microscopy revealed that 392 cells (49.0%) were singlets while the remaining cells were doublets (6.75%) or empty wells. Only single cells were subsequently analyzed. After extensive quality checking of cells based on the number of detected genes, total read counts and expression of mitochondrial genes, 92 cells were selected for final analysis (Supplemental Figure S3).

### 3.2. Unsupervised UMAP Divided Cells into Six Clusters

**Figure 2:**
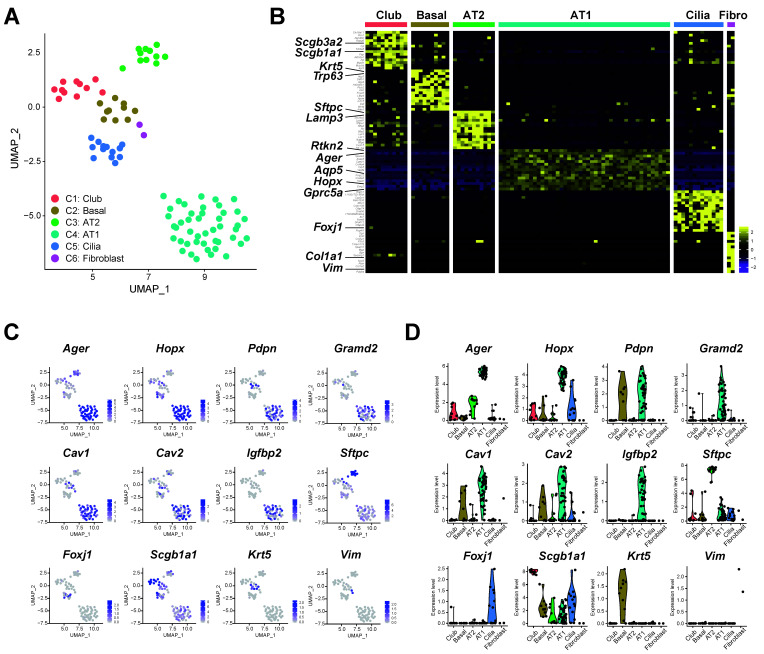
Clustering of 92 tdTomato-positive cells in *ACID;R26tdTomato* mice. (**A**) Unsupervised UMAP divided 92 cells into 6 clusters (C1–C6). Expression level (C and D) represents absolute read count. (**B**) Heatmap with 10–15 markers representative of each cell type. Yellow and purple colors indicate high and low expression, respectively. (**C**) UMAP plots with known cell type-specific marker genes. AT1 cells (*Ager*, *Hopx*, *Pdpn*, *Igfbp2*, *Cav1*, *Cav2* and *Gramd2*), AT2 cells (*Sftpc*), club cells (*Scgb1a1*), basal cells (*Krt5*), ciliated cells (*Foxj1*) and fibroblasts (*Vim*). Blue and grey indicate high and low expression, respectively. (**D**) Violin plot with the same marker genes as in C.

**Figure 3:**
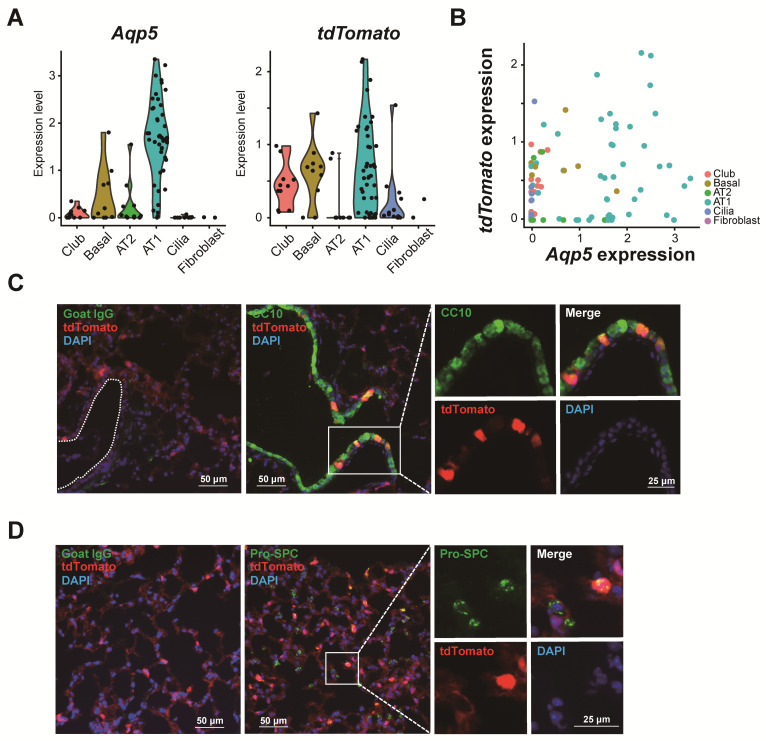
TdTomato expression in *ACID;R26tdTomato* mouse lung. (**A**) Violin plot for *Aqp5* (left) and *tdTomato* (right). (**B**) Scatter plot between *Aqp5* and *tdTomato* expression. (**C**) Immunofluorescence staining shows CC10 (green) and tdTomato (red) expression in *ACID;R26tdTomato* mouse lung. Goat IgG (left panel) was used as negative control. White dotted line indicates outline of airway. (**D**) Immunofluorescence staining shows pro-SP-C (green) and tdTomato (red) expression in *ACID;R26tdTomato* mouse lung. Goat IgG (left panel) was used as negative control.

Unsupervised UMAP divided these 92 cells into six clusters (C1–C6, Figure 2A), and enriched genes in each cluster were identified (Supplementary Table S1). A heatmap with 10–15 markers representative of fibroblasts, club, basal, AT2, AT1 and ciliated cells showed that gene expression patterns of these six clusters correspond to specific cell types (Figure 2B). For C6, the number of fibroblasts was small (*N* = 2) so that no C6-enriched genes met statistical significance; however, the cluster was distinct from the others as shown by the heat map (Figure 2B). As expected, AT1 cells represented the biggest cluster (Figure 2A, cluster C4). The expression of various cell-specific markers confirmed that C1 represented club cells (*Scgb1a1*), C2 basal cells (*Krt5*), C3 AT2 cells (*Sftpc*), C4 AT1 cells (*Ager*, *Hopx*, *Pdpn*, *Cav1*, *Cav2*, *Igfbp2* and *Gramd2*) and C5 ciliated cells (*Foxj1*), as shown in the UMAP (Figure 2C) and violin plots (Figure 2D). Of note, the expression of endogenous markers (e.g., beta-actin, *Actb*) was stable among cell clusters (Supplemental Figure S4). The full list of enriched genes in each cluster is reported in Supplemental Table S1. The expression of *tdTomato* most closely resembled that of *Aqp5* (Figure 3A), and both *tdTomato*^+^ and *Aqp5*^+^ cells were highly enriched in the AT1 cell population. A moderate correlation between *Aqp5* and *tdTomato* expression was observed for epithelial cells (R = 0.32, *p* < 0.05, Figure 3B). Immunofluorescence staining of mouse lung tissue showed that ~10% of tdTomato^+^ cells were also labeled with CC10 (Figure 3C) and about 12% with pro-SPC (Figure 3D), indicating tdTomato expression in some club and AT2 cells in the *ACID;R26tdTomato* mouse, although correlation between *Aqp5* and *Scgb1a1* and *Sftpc* expression was very weak (R = −0.06 and −0.15, respectively). Nevertheless, the largest cluster was identified as AT1 cells, indicating that tdTomato sorting of lung cells isolated from *ACID;R26tdTomato* mice successfully enriched for the AT1 cell population.

### 3.3. Identification of Gprc5a as a Novel Candidate AT1 Cell Marker

Our scRNA-seq data indicated that some known AT1 cell genes such as *Pdpn*, *Cav1* and *Cav2* are also variably expressed at low levels in airway epithelial cells and AT2 cells (Figure 2C,D, Supplemental Figure S5A), reinforcing the notion that specificity of currently available AT1 cell markers is not optimal. Furthermore, three of these classical markers (*Pdpn*, *Aqp5* and *Cav2*), as well as other more recently described markers (e.g., *Scnn1g*, *Gramd2* and *Igfbp2*), were found to be heterogeneously expressed in subsets of AT1 cells at the single-cell level (Supplemental Figure S5B).

**Figure 4:**
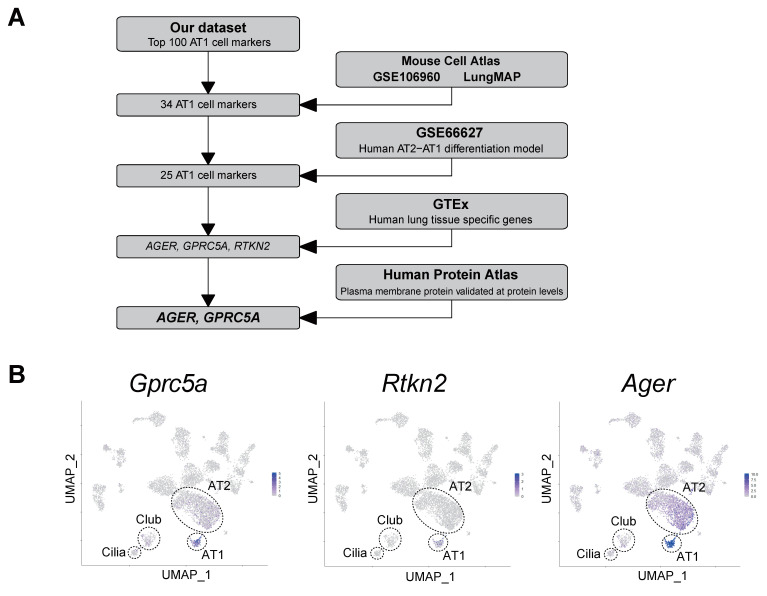

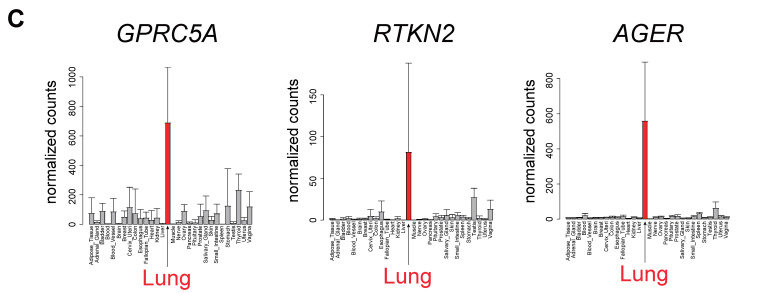
High expression of *Gprc5a*, *Rtkn2* and *Ager* in AT1 cells and lung. (**A**) Flowchart for identifying AT1 cell- and lung-enriched genes. (**B**) UMAP of scRNA-seq data from Mouse Cell Atlas [28] with *Gprc5a*, *Rtkn2* and *Ager*. Blue and grey indicate high and low expression (determined by absolute read count), respectively. (**C**) Gene expression of *GPRC5A*, *RTKN2* and *AGER* in lung (red) and 29 other organs (grey) from GTEx database. Counts per million was used for normalization between samples.

Our scRNA-seq was purposely biased by the use of Aqp5-lineage traced mice, limiting the analysis to only the Aqp5-positive AT1 cell population, and was used as a way to enrich for these cells. Subsequently, in order to identify a novel candidate AT1 cell surface marker, we undertook an unbiased approach, shown schematically in Figure 4A, by integrating our data with existing datasets reporting gene expression in AT1 cells. Because our goal was to identify a surface marker conserved between murine and human species, we included in our analysis available RNA-seq datasets originating from both mouse and human samples. First, the top 100 genes with the lowest false discovery rate (FDR) values and highest fold-change in AT1 cells were extracted from our dataset (Supplemental Table S2). We then integrated these 100 genes with three independent scRNA-seq datasets of mouse lung in which AT1 cell markers were included [28,30,41], further reducing the number of AT1 cell-enriched genes to 34 (“AT1 cell-enriched among all datasets” column in Supplemental Table S2). Next, we compared these data with RNA-seq data from our in vitro human AT2-AT1 cell differentiation model [12]. Among 34 genes, 25 were also enriched in AT1-like cells. Of note, *Gramd2* which was recently identified by cross-species transcriptome profiling between human and rat [12] is included in this list (Supplemental Table S2). Furthermore, *Gprc5a*, which was suggested as a possible AT1 cell marker in the same study but not further investigated, is in the list as well. Subsequently, to ensure that the candidate AT1 cell marker was not expressed in other tissues, we extracted lung-enriched genes using GTEx, which contains RNA-seq data of human tissues from various organs. Among the 25 genes, *Rtkn2*, *Ager*, and *Gprc5a* were identified as both AT1 cell- and lung-tissue enriched genes. UMAP plot from the Mouse Cell Atlas shows that these three genes are enriched in AT1 cells, although *Ager* is highly expressed in AT2 cells and *Gprc5a* is slightly expressed in some AT2 cells (Figure 4B). UMAP plots for ‘classical’ AT1 cell markers are shown in Supplemental Figure S6. On the other hand, *Rtkn2* appears to be expressed in only a subset of AT1 cells (Figure 4B). RNA-seq data from GTEx show that expression of these three genes in lung is clearly higher than in other organs (Figure 4C). Finally, integrating the comprehensive annotation of protein localization from the Human Protein Atlas, GPRC5A and AGER, but not RTKN2, were found to be plasma membrane proteins already validated at the protein level, and specifically GPRC5A protein was reported as ‘high in pneumocytes’ (https://www.proteinatlas.org/). We selected GPRC5A for further validation as it strictly met the following criteria: (1) mouse/human conservation, (2) lung specificity, (3) AT1 cell enrichment and 4) transmembrane localization criteria. This made it the best novel AT1 cell marker candidate with high potential for future isolation of AT1 cells and generation of specific Cre reporter mice.

### 3.4. Immunofluorescence Staining Revealed that GPRC5A is Specifically Expressed in Mouse, Rat and Human AT1 Cells

**Figure 5:**
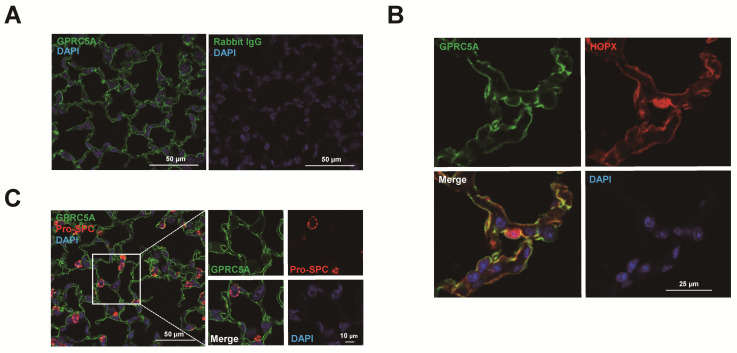
GPRC5A expression in mouse distal lung. (**A**) Representative single staining for GPRC5A (green) in mouse distal lung. Blue = DAPI. Rabbit IgG (right panel) was used as negative control. (**B**) Representative double staining for GPRC5A (green) and HOPX (red) in mouse distal lung. Blue = 4′,6-diamidino-2-phenylindole (DAPI). Negative controls are shown in Supplemental Figure S7A. (**C**) Representative double staining for GPRC5A (green) and pro-SPC (red) in mouse distal lung. Blue = DAPI. Negative controls are shown in Supplemental Figure S7B.

**Figure 6:**
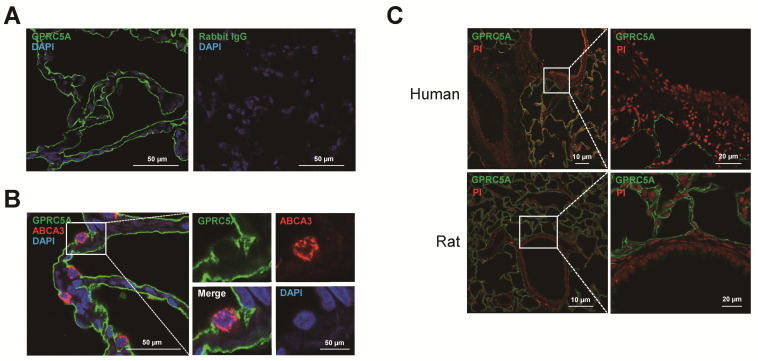
GPRC5A expression in human and rat lung. (**A**) Representative single staining for GPRC5A (green) in human distal lung. Blue = DAPI. Rabbit IgG (right panel) is the negative control. (**B**) Representative double staining for GPRC5A (green) and ABCA3 (red) in human distal lung. Blue = DAPI. Negative controls are showed in Supplemental Figure S7C. (**C**) Representative single staining for GPRC5A (green) in human (upper panel) and rat (lower panel) airway. White box indicates airway. Red = propidium iodide (PI).

**Figure 7:**
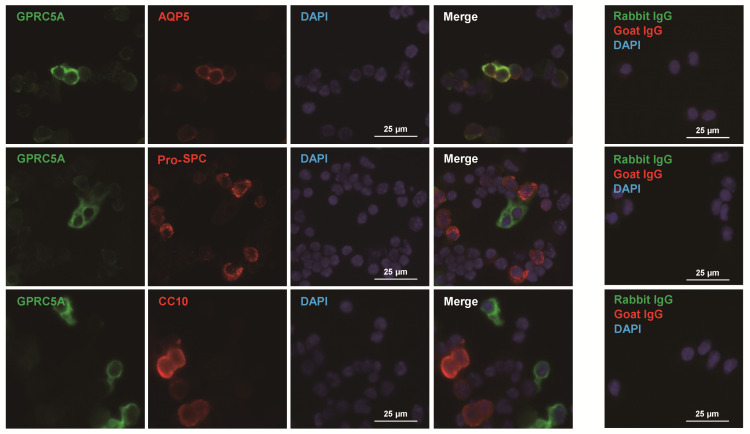
Co-staining of GPRC5A with AQP5, pro-SPC and CC10 in crude cytospin preparations from mouse lung. Representative double staining for GPRC5A (green) and AQP5 (upper panel, red), pro-SPC (middle panel, red) or CC10 (lower panel, red) in cytospins of crude preparations from digested mouse lung tissue. Blue = DAPI. Rabbit and goat IgG were negative controls.

To confirm GPRC5A protein expression in AT1 cells, immunofluorescence staining was performed. GPRC5A was localized at the surface of AT1 cells in mouse lung (Figure 5A), and double staining with HOPX showed clear co-localization of GPRC5A and HOPX (Figure 5B). Negative controls are shown in Supplemental Figure S7A. Furthermore, double staining with pro-SPC showed that AT2 cells do not express GPRC5A (Figure 5C), indicating that expression of GPRC5A protein is AT1 cell-specific in mouse alveoli. Negative controls are shown in Supplemental Figure S7B. Next, we performed immunofluorescence with human lung tissue. Similar to findings in mouse lung, GPRC5A is clearly localized at the surface of AT1 cells (Figure 6A) while human AT2 cells, which are positive for ABCA3, do not express GPRC5A (Figure 6B). Negative controls are shown in Supplemental Figure S7C. Proximal and distal mouse, human and rat airways did not express GPRC5A above background (Figure 6C and Supplemental Figure S8). AT1 cell-specific staining of GPRC5A was also confirmed by immunofluorescence co-staining with AQP5, pro-SPC and CC10 in cytospins of digested mouse lung (Figure 7). Collectively, these results confirm that GPRC5A protein is specifically expressed on the surface of AT1 cells in both mouse and human lungs.

## 4. Discussion

We successfully performed scRNA-seq of FACS-sorted tdTomato^+^ AT1 cells from *ACID;R26tdTomato* reporter mice and integrated these results with publicly available mouse and human datasets, with the goal of identifying and further characterizing novel AT1 cell genes that could also be used as surface markers to facilitate cell sorting. In our scRNA-seq results, approximately 50% of the 92 cells analyzed expressed conventional AT1 cell markers, while smaller subsets expressed markers of other cell types such as those for AT2, club, basal or ciliated cells, indicating that this approach allowed considerable enrichment for AT1 cells. Among the frequently used ‘classical’ AT1 cell markers, we found that *Pdpn*, *Cav1*, *Cav2* and *Aqp5* were also expressed in some cells in other epithelial clusters, and that *Pdpn*, *Cav2*, *Scnn1g*, *Gramd2*, *Igfbp2* and *Aqp5* were heterogeneously expressed within the AT1 cell population at a single-cell level. By integrating our scRNA-seq data with public datasets and our previous in vitro AT2 to AT1 cell differentiation model dataset [10,12,28,30], we overcame the bias of analyzing only Aqp5-positive cells and identified three robust putative AT1 cell-enriched and lung-specific genes: *Rtkn2*, *Ager* and *Gprc5a*. Based on strict selection to fulfill criteria of mouse/human conservation, AT1 cell-enriched expression and membrane localization, we selected *Gprc5a* as the best candidate for further characterization. Consistent with our selection, immunofluorescence staining of human, mouse and rat lung sections validated GPRC5A as a candidate AT1 cell surface marker. It is important to note that neither GPRC5A nor previously identified AT1 markers are uniquely expressed in AT1 cells, and further studies are needed to determine whether this reflects the biological function or origin of AT1 cells. Nevertheless, the identification of a conserved surface marker enriched in the AT1 cell population, such as GPRC5A, offers a novel tool for further study and characterization of AT1 cells.

Within our dataset, airway cell clusters in the *ACID;R26tdTomato* mouse were also positive for *tdTomato* (Figure 3A), with immunofluorescence staining showing that ~10% of CC10^+^ cells are tdTomato^+^ (Figure 3C). This is consistent with our previous study which revealed that *Aqp5* is also expressed in trachea and in a subset of conducting airway epithelial cells, as shown in *ACID;R26LacZ^+/+^* mice [25]. Accordingly, we found moderate, but not strong, correlation between *Aqp5* and *tdTomato* expression (Figure 3B), suggesting that some *tdTomato*-positive cells do not express *Aqp5* and conversely that not all *Aqp5*-expressing cells express *tdTomato*. Given that Cre is constitutively expressed, expression of *tdTomato* in *Aqp5*^−^ cells may be the result of recombination during development with subsequent loss of *Aqp5*.

Regardless, these findings indicate that *ACID;R26tdTomato* mice can be used to enrich for AT1 cells for subsequent downstream analyses by sorting for tdTomato^+^ cells, which we will pursue in future studies. Here, we also found that some *tdTomato*^+^ cells express AT2 cell markers, and some cells expressing AT2 cell markers also express *Aqp5* (Figure 3A). This could reflect true expression of *Aqp5* in AT2 cells, or the presence of cells intermediate between AT1 and AT2 cells as we and others have previously shown [22,27]. However, in the present study, the number of potential intermediate cells was too small for further characterization.

Despite limitations of the Fluidigm system with regard to capturing only small numbers of cells, a major strength is the greater depth of sequencing compared to other methods. For example, a recent study identified a novel subpopulation of pulmonary neuroendocrine cells showing that a small number of cells can lead to significant insights [42]. This enabled us to demonstrate heterogeneity among AT1 cells with regard to the expression of AT1 cell markers (Supplementary Figure S5B). Interestingly, we found that some well-known AT1 cell markers, such as *Pdpn*, *Igfbp2*, *Cav2* or *Gramd2*, were not uniformly expressed in all AT1 cells (Supplemental Figure S5B), indicating considerable heterogeneity among AT1 cells within this cluster. This is consistent with other published findings of AT1 cell sub-populations and may also reflect the fact that AT1 cell marker expression can change over time [27,43], so cells that are positive for one marker might later become positive/negative for another marker. Further studies and a collaborative effort to analyze and compare datasets generated from different research groups in the field is needed to clarify and fully understand the basis for marker heterogeneity in AT1 cells. Our observation that *Igfbp2* is expressed in only half of the *Aqp5*^+^ AT1 cells (Supplementary Figure S5B) is in conflict with previous findings showing that most (95%) *Hopx*^+^ AT1 cells isolated from adult (postnatal day 60) *Sftpc-CreER;Rosa26-Zsgreen;Hopx-tdTomato* mice express *Igfbp2* [30]. This discrepancy may be due to a different sorting strategy that led to isolation of different cell populations or strain differences.

In our scRNA-seq data, the percentage of mitochondrial genes is relatively high (Supplementary Figure S3), suggesting that enzymatic digestion and the FACS procedure are still quite harsh for the relatively fragile AT1 cells. Other approaches such as single-nuclei RNA-seq (snRNA-seq), in which mild digestion can be done to reduce damage to AT1 cell nuclei, may be an alternative option to study gene expression of AT1 cells, as recently reported [44].

The isolation and characterization of AT1 cells represents a major challenge within the pulmonary field, due to both the heterogeneity and lack of specificity of expression of AT1 cell markers, as well as the lack of robust antibodies for their detection. By integrating several datasets, we identified three putative AT1 cell-enriched and lung-specific genes: *Gprc5a*, *Rtkn2* and *Ager.* Among these three putative AT1 markers, *Rtkn2* expression appears as the most ‘AT1-specific’. However, while *Gprc5a* and *Ager* are expressed in almost all AT1 cells (Figure 4B), *Rtkn2 is* restricted to only a subset of AT1 cells, and it would not be a useful surface marker for AT1 cell isolation as it encodes for a nuclear protein. Because of its potential usefulness as a surface marker for isolation of AT1 cells, we chose the transmembrane protein GPRC5A for further validation. Although there is low-level expression of *Gprc5a* in mouse (Figure 4B) and human AT2 cells [21,23] at the mRNA level, as well as in bronchial airway epithelial cells [23], we found that at the protein level GPRC5A is very specific for AT1 cells in mouse, rat and human lungs (Figure 5 and Figure 6 and Supplemental Figure S8), despite a previous report of expression in airway epithelial cells [45]. We show that it is localized to the membrane of AT1, but not AT2 or airway cells by immunostaining, supporting its value as a highly promising surface marker for sorting AT1 cells specifically from distal lung cell populations.

*Gprc5a* was first cloned as a retinoic acid-inducible gene 1 (RAIG1) which has a sequence containing seven transmembrane domains, characteristic of G protein–coupled receptors [46]. *Gprc5a* knockout mice do not show developmental abnormalities or phenotypic changes in the lung, but develop spontaneous lung adenocarcinomas [47]. Deletion of this gene confers susceptibility to endotoxin-induced pulmonary edema and injury [48], indicating that GPRC5A is critical for lung homeostasis and functions as a tumor suppressor. Consistent with this, GPRC5A expression was found to be decreased in patients with chronic obstructive pulmonary disease (COPD) and non-small-cell lung cancer [45]. GPRC5A was shown to transduce NF-κB and STAT3 signaling [49], but the molecular function of GPRC5A, especially in AT1 cells, has not been elucidated to date. Interestingly, we previously showed a role for retinoid X receptor (RXR) signaling in AT2 to AT1 cell differentiation in vitro [10], suggesting a mechanism whereby GPRC5A might be upregulated during this phenotypic transition. Further characterization of the role of GPRC5A in AT1 cells is needed.

In summary, using *ACID;R26tdTomato* mice, we enriched for AT1 cells and successfully performed scRNA-seq, an analysis which has previously been limited by the difficulty in isolating adequate numbers of viable AT1 cells. We show that at the single-cell level, many frequently used AT1 cell markers lack specificity. Heterogeneity in expression of putative AT1 cell markers was observed, emphasizing the need for development of additional AT1 cell (and especially surface) markers for more complete characterization and sorting of the entire population of AT1 cells. Our approach allowed sufficient enrichment of AT1 cells to enable identification and characterization of GPRC5A as a potential novel validated surface marker for AT1 cells. Analysis of greater numbers of AT1 cells is warranted to validate and further characterize subgroups of AT1 cells as well as novel AT1 cell functions.

## Supplementary Materials

The following are available online at https://www.mdpi.com/2073-4409/9/11/2460/s1, Figure S1: Generation of *ACID; R26tdTomato* mice. Figure S2: Negative controls for gating for FACS. Figure S3: Quality checking of cells after sequencing. Figure S4: Expression of an endogenous control in our scRNA-seq data. Figure S5: Expression of conventional AT1 cell markers in non-AT1 cell epithelial clusters and heterogeneous expression in AT1 cells. Figure S6: Expression of representative AT1 cell markers in mouse AT1 cells. Figure S7: Negative controls for immunofluorescence. Figure S8: GPRC5A expression is not detected in mouse and human proximal airways. Table S1: Enriched genes in each cluster. Table S2: Top 100 AT1 cell-enriched genes in our scRNA-seq analysis.
