# Supplementary material for: Integrated Single-Cell RNA-Sequencing Analysis of Aquaporin 5-Expressing Mouse Lung Epithelial Cells Identifies GPRC5A as a Novel Validated Type I Cell Surface Marker
Source: Cells. 2020 Nov 11;9(11):2460. doi: 10.3390/cells9112460 (PMC7697677; doi:10.3390/cells9112460)
Supplement: Supplementary file 1 [file cells-09-02460-s001.zip › 2020-11-09_New Suppl/Horie-Castaldi et al_new Supplementary Figure S2.pdf]

Supplemental Figure S2

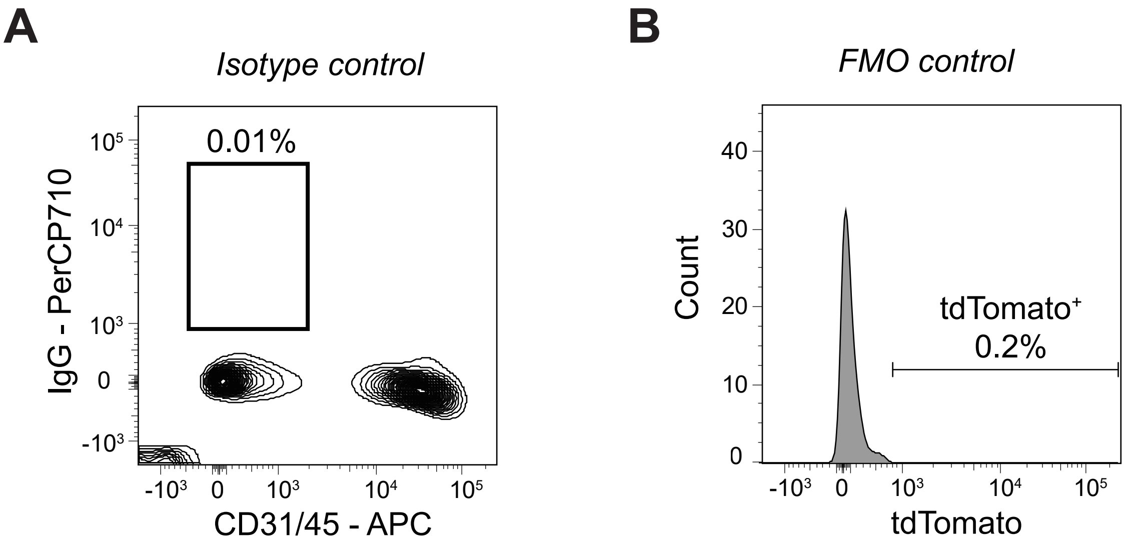

Supplemental Figure S2. Negative controls for gating for FACS.
A) Isotype control for E-cadherin-PerCP710. B) Fluorescence Minus One (FMO) control for tdTomato.
