# Supplementary material for: Integrated Single-Cell RNA-Sequencing Analysis of Aquaporin 5-Expressing Mouse Lung Epithelial Cells Identifies GPRC5A as a Novel Validated Type I Cell Surface Marker
Source: Cells. 2020 Nov 11;9(11):2460. doi: 10.3390/cells9112460 (PMC7697677; doi:10.3390/cells9112460)
Supplement: Supplementary file 1 [file cells-09-02460-s001.zip › 2020-11-09_New Suppl/Horie-Castaldi et al_new Supplementary Figure S3.pdf]

Supplemental Figure S3

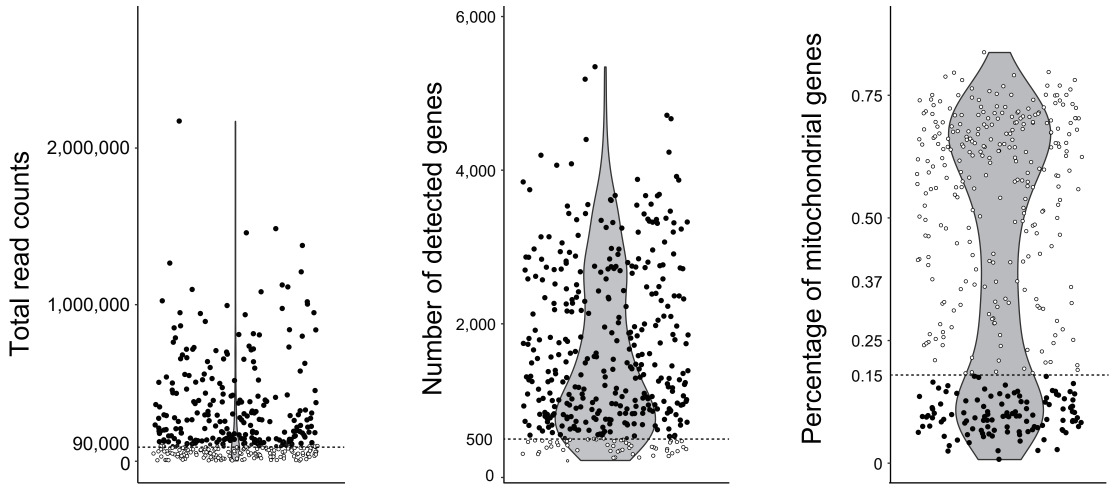

Supplemental Figure S3. Quality checking of cells after sequencing.
Left: Violin plot of total read counts (cut off: 90,000). Middle: Violin plot of detected genes (cut off: 500). Right: Violin plot of proportion of sequence reads from mitochondrial genes (cut off: 0.15). Dots indicate each cell and black dots indicate cells which passed the cutoff values for each parameter.
