# Supplementary material for: Integrated Single-Cell RNA-Sequencing Analysis of Aquaporin 5-Expressing Mouse Lung Epithelial Cells Identifies GPRC5A as a Novel Validated Type I Cell Surface Marker
Source: Cells. 2020 Nov 11;9(11):2460. doi: 10.3390/cells9112460 (PMC7697677; doi:10.3390/cells9112460)
Supplement: Supplementary file 1 [file cells-09-02460-s001.zip › 2020-11-09_New Suppl/Horie-Castaldi et al_new Supplementary Figure S5.pdf]

Supplemental Figure S5

A

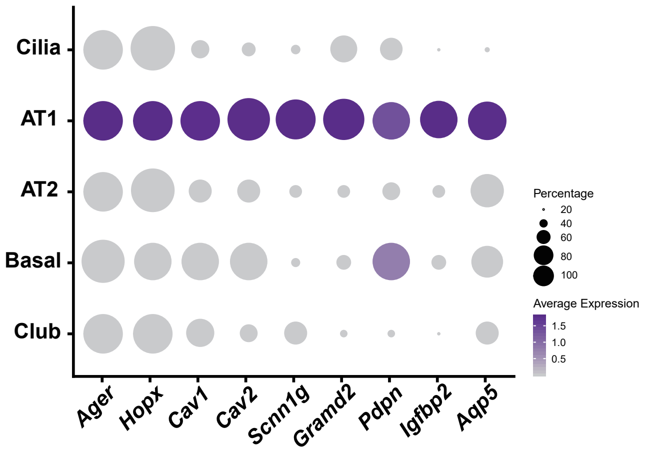

B

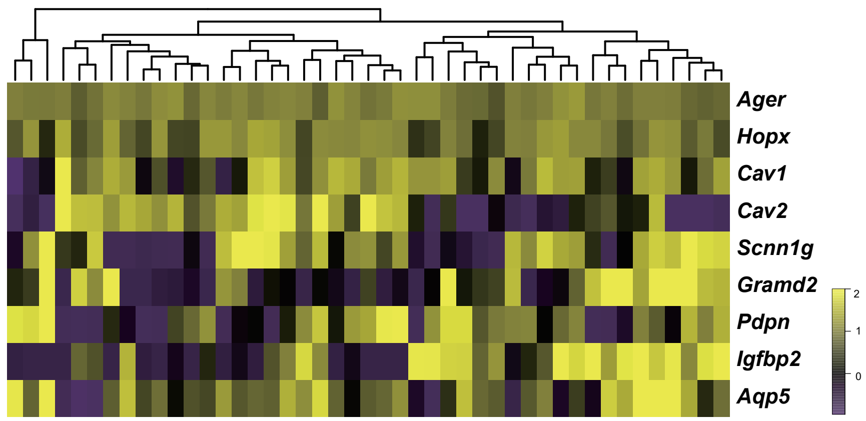

Supplementary Figure S5. Expression of conventional AT1 cell markers in non-AT1 cell epithelial clusters and heterogeneous expression in AT1 cells.

A: Dot plot of percent of cells expressing conventional AT1 cell markers (*Ager, Hopx, Cav1, Cav2, Scnn1g, Gramd2, Pdpn, Igfbp2* and *Aqp5*, dot size) and average expression (color scale) in each epithelial cluster.

B: Heatmap of 45 AT1 cells with conventional or frequently used AT1 cell markers (*Ager, Hopx, Cav1, Cav2, Scnn1g, Gramd2, Pdpn, Igfbp2* and *Aqp5*). Yellow and purple indicate high and low expression, respectively.
