# Supplementary material for: Integrated Single-Cell RNA-Sequencing Analysis of Aquaporin 5-Expressing Mouse Lung Epithelial Cells Identifies GPRC5A as a Novel Validated Type I Cell Surface Marker
Source: Cells. 2020 Nov 11;9(11):2460. doi: 10.3390/cells9112460 (PMC7697677; doi:10.3390/cells9112460)
Supplement: Supplementary file 1 [file cells-09-02460-s001.zip › 2020-11-09_New Suppl/Horie-Castaldi et al_new Supplementary Figure S6.pdf]

Supplemental Figure S6

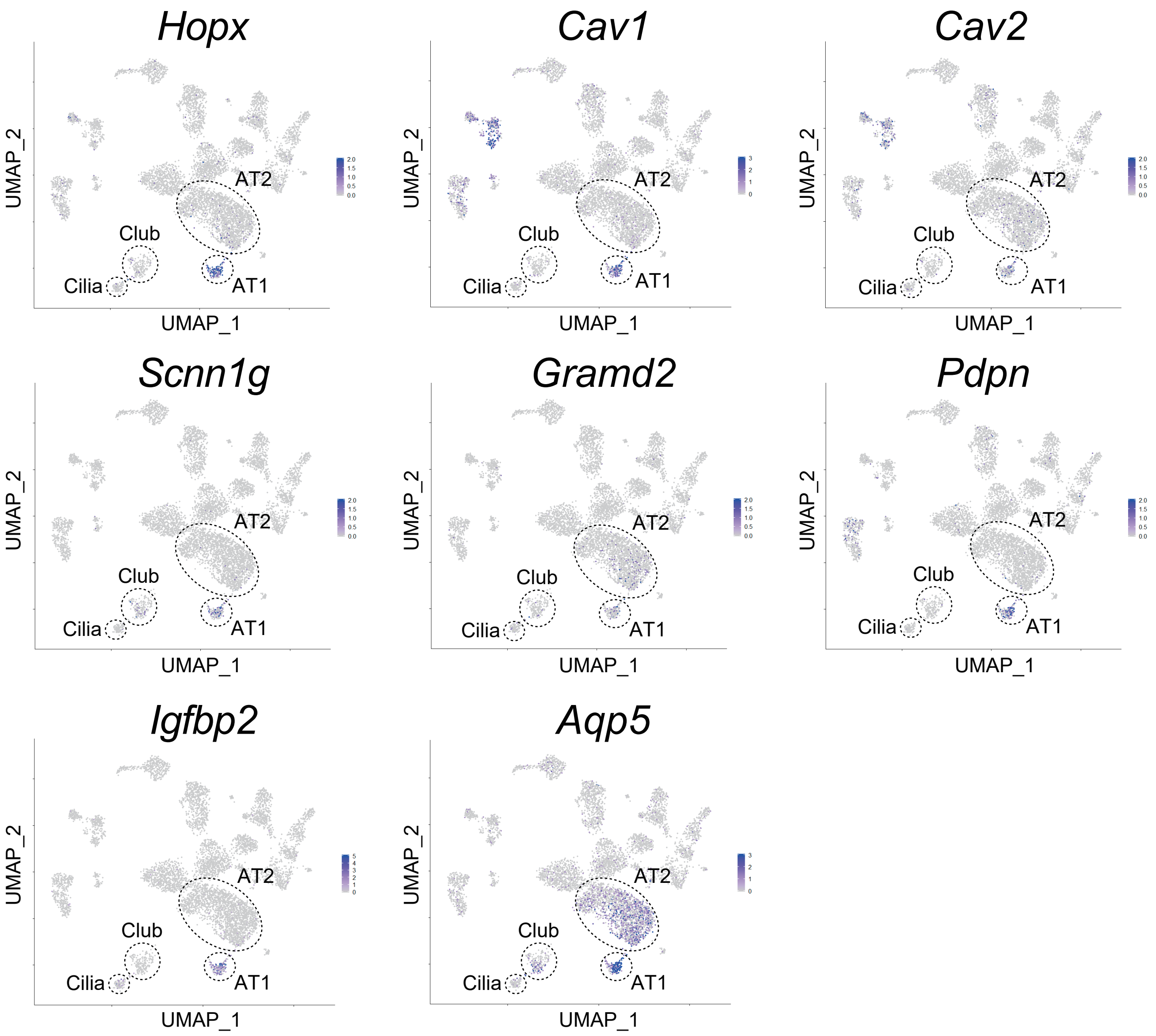

Supplementary Figure S6. Expression of representative AT1 cell markers in mouse AT1 cells.
UMAP of scRNA-seq data from Mouse Cell Atlas (Han X, *et al.*) with *Hopx, Cav1, Cav2, Scnn1g, Gramd2, Pdpn, Igfbp2* and *Aqp5*. Blue and grey indicate high and low expression, respectively.
