# Supplementary material for: Integrated Single-Cell RNA-Sequencing Analysis of Aquaporin 5-Expressing Mouse Lung Epithelial Cells Identifies GPRC5A as a Novel Validated Type I Cell Surface Marker
Source: Cells. 2020 Nov 11;9(11):2460. doi: 10.3390/cells9112460 (PMC7697677; doi:10.3390/cells9112460)
Supplement: Supplementary file 1 [file cells-09-02460-s001.zip › 2020-11-09_New Suppl/Horie-Castaldi et al_NEW Supplementary Figure S7.pdf]

Supplemental Figure S7

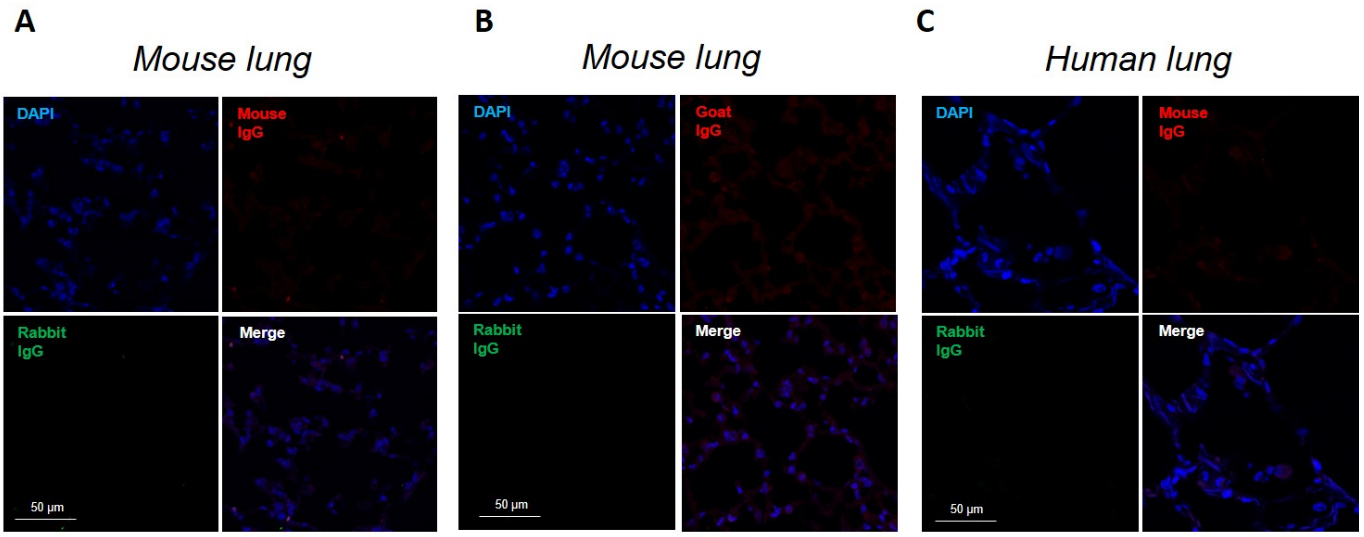

Supplemental Figure S7. Negative controls for immunofluorescence.
Normal rabbit IgG is the negative control for GPRC5A in mouse and human lung sections in Figures 5B and C and Figure 6 B. A) Mouse lung section. Normal mouse IgG is negative control for HOPX (Figure 5 B, in the manuscript). B) Mouse lung section. Normal goat IgG is negative control for pro-SPC (Figure 5 C, in the manuscript). C) Human lung section. Normal mouse IgG is negative control for ABCA3 (Figure 6 B, in the manuscript).
