# Supplementary material for: Integrated Single-Cell RNA-Sequencing Analysis of Aquaporin 5-Expressing Mouse Lung Epithelial Cells Identifies GPRC5A as a Novel Validated Type I Cell Surface Marker
Source: Cells. 2020 Nov 11;9(11):2460. doi: 10.3390/cells9112460 (PMC7697677; doi:10.3390/cells9112460)
Supplement: Supplementary file 1 [file cells-09-02460-s001.zip › 2020-11-09_New Suppl/Horie-Castaldi et al_NEW Supplementary Figure S8.pdf]

Supplemental Figure S8

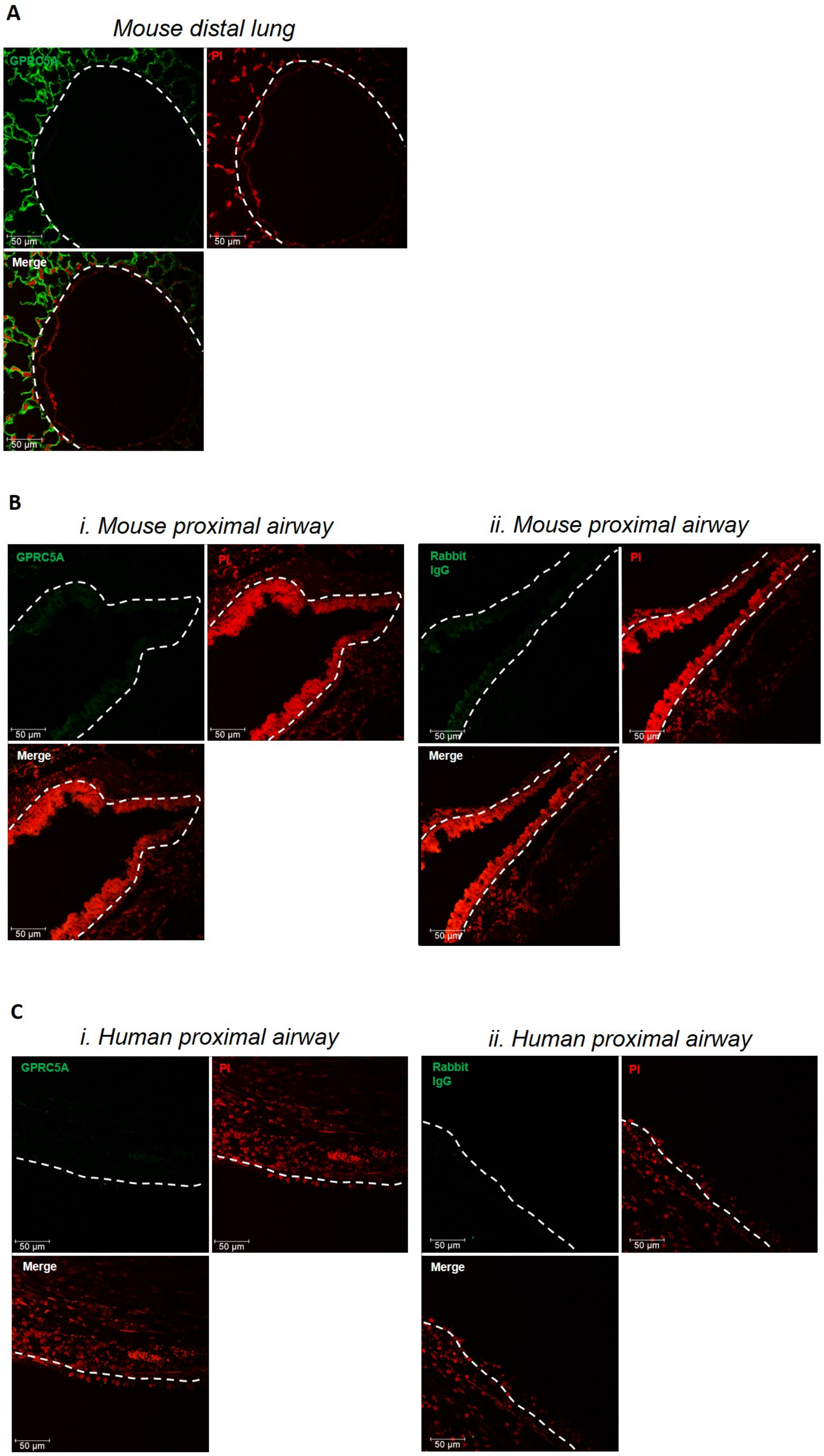

Supplemental Figure S8. GPRC5A expression is not detected in mouse and human proximal airways. Green = GPRC5A or normal rabbit IgG for negative control. Red = propidium iodide (PI). A) Mouse distal lung section used as positive control. B) Mouse proximal airway (i) GPRC5A and (ii) negative control; representative images for n = 3. C) Human proximal airway (i) GPRC5A and (ii) negative control; n = 1.
