# Supplementary material for: Integrated Single-Cell RNA-Sequencing Analysis of Aquaporin 5-Expressing Mouse Lung Epithelial Cells Identifies GPRC5A as a Novel Validated Type I Cell Surface Marker
Source: Cells. 2020 Nov 11;9(11):2460. doi: 10.3390/cells9112460 (PMC7697677; doi:10.3390/cells9112460)
Supplement: Supplementary file 1 [file cells-09-02460-s001.zip › 2020-11-09_New Suppl/Horie-Castaldi et al_Supplementary Figure S1.pdf]

Supplemental Figure S1

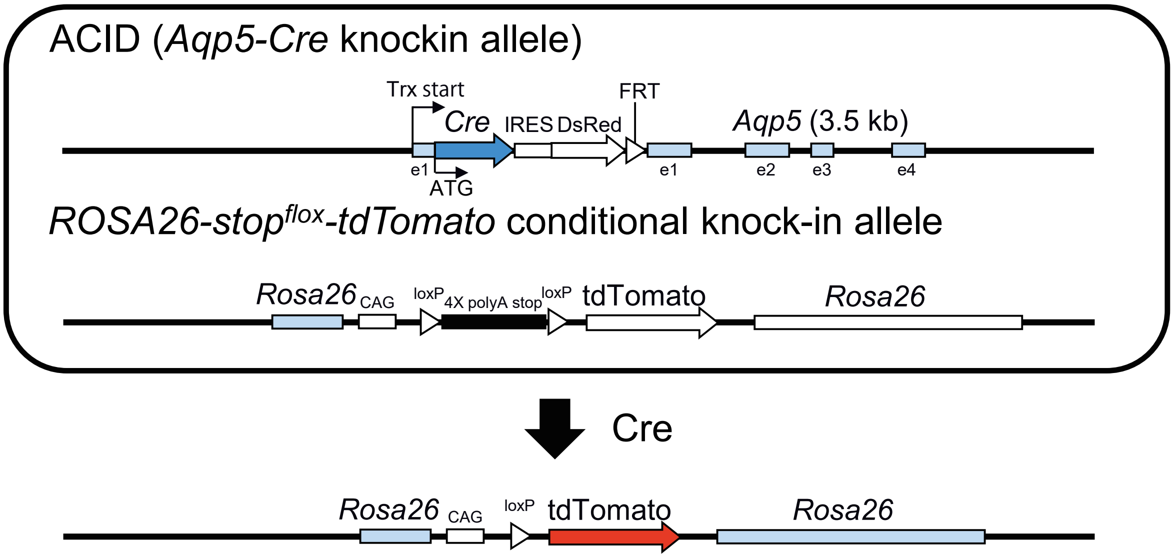

Supplemental Figure S1. Generation of *ACID;R26tdTomato* mice.
Aqp5-Cre-IRES-DsRed (ACID) mice in which a Cre-IRES-DsRed cassette is knocked into exon 1 of the endogenous *Aqp5* gene were crossed to mice with a *ROSA26-stopflox-tdTomato* conditional knockin allele. Double-heterozygous mice (termed *ACID;R26tdTomato*) which express the red fluorescent protein Tomato after Cre/loxP recombination were generated.
